# Expression of aquaporin1, a water channel protein, in cytoplasm is negatively correlated with prognosis of breast cancer patients

**DOI:** 10.18632/oncotarget.6994

**Published:** 2016-01-23

**Authors:** Fengxia Qin, Huikun Zhang, Ying Shao, Xiaoli Liu, Limin Yang, Yong Huang, Li Fu, Feng Gu, Yongjie Ma

**Affiliations:** ^1^ Department of Breast Cancer Pathology and Research Laboratory, Key Laboratory of Breast Cancer Prevention and Therapy (Ministry of Education), National Clinical Research Center for Cancer, Tianjin, China; ^2^ Department of Tumor Cell Biology, Key Laboratory of Cancer Prevention and Therapy of Tianjin, Tianjin Medical University Cancer Institute and Hospital, National Clinical Research Center for Cancer, Tianjin, China

**Keywords:** aquaporin1, breast cancer, prognosis, metastasis, cytoplasm

## Abstract

Aquaporin1 (AQP1) belongs to a highly conserved family of aquaporin proteins which facilitate water flux across cell membranes. Although emerging evidences indicated the cytoplasm was important for AQP1 localization, the function of AQP1 corresponding to its cytoplasmic distribution has rarely been explored until present. In our clinical study, we reported for the first time that AQP1 was localized dominantly in the cytoplasm of cancer cells of invasive breast cancer patients and cytoplasmic AQP1 was an independent prognostic factor. High expression of AQP1 indicated a shorter survival, especially in luminal subtype. Moreover, in line with our findings in clinic, cytoplasmic expression of AQP1 was further validated in both primary cultured breast cancer cells and AQP1 over-expressing cell lines, in which the functional importance of cytoplasmic AQP1 was confirmed *in vitro*. In conclusion, our study provided the first evidence that cytoplasmic expression of AQP1 promoted breast cancer progression and it could be a potential prognostic biomarker for breast cancer.

## INTRODUCTION

Aquaporins (AQPs) are a family of channel-forming glycoproteins which function mostly as semi-selective pores facilitating water transport in response to osmotic and hydrostatic differences [1, 2]. Aquaporin1 (AQP1) was initially identified on the cell membranes of erythrocytes in 1988 and its classical role in facilitating transcellular water movement has been extensively studied and well understood [3–8]. Subsequent analysis has revealed that AQP1 serves as more than a water channel. Its involvement in cell migration, fat metabolism, leukocyte biology and neural signal transduction indicated its important role in the pathophysiology of cancer, obesity, immune cell dysfunction and epilepsy [9–12].

Clinical evidence suggested that up-regulation of AQP1 was observed in a variety of malignancies such as brain tumors, cervical carcinoma, and colon tumors and high expression of AQP1 promoted tumor progression [13–18]. Until present, all previous studies were focused on its function of membranous expression. Actually, AQP1 was also reported to be massively distributed throughout the cytoplasm in primary rat astrocytes detected by immunocytochemistry analysis [19]. Both LaRusso's and Bill's groups demonstrated that AQP1 was observed in cytoplasm of normal and tumor cells and could translocate to cell membrane after certain kind of stimulation [20, 21]. Furthermore, Monzani et al. found that AQP1 could bind with Lin-7 and contributed to cell migration through Lin7/β-catenin interaction [22]. All these studies demonstrated that cytoplasm was important for AQP1 localization beside of cell membrane. However, there were almost no reports focused on the function of AQP1 corresponding to its cytoplasmic distribution.

In our present study, we used a large cohort of human invasive breast cancer specimens to investigate the expression and function of AQP1. Completely different from the membranous expression of AQP1 in myoepithelial cells of ducts in breast benign lesions and ductal carcinoma *in situ* (DCIS), we reported for the first time that AQP1 exhibited cytoplasmic expression pattern in cancer cells of invasive ductal carcinoma (IDC). Furthermore, we demonstrated firstly that high expression of AQP1 indicated a poor prognosis and cytoplasmic expression of AQP1 was an independent prognostic factor of IDC patients.

## RESULTS

### AQP1 exhibited distinct cellular localization in different breast tissues

**Figure 1:**
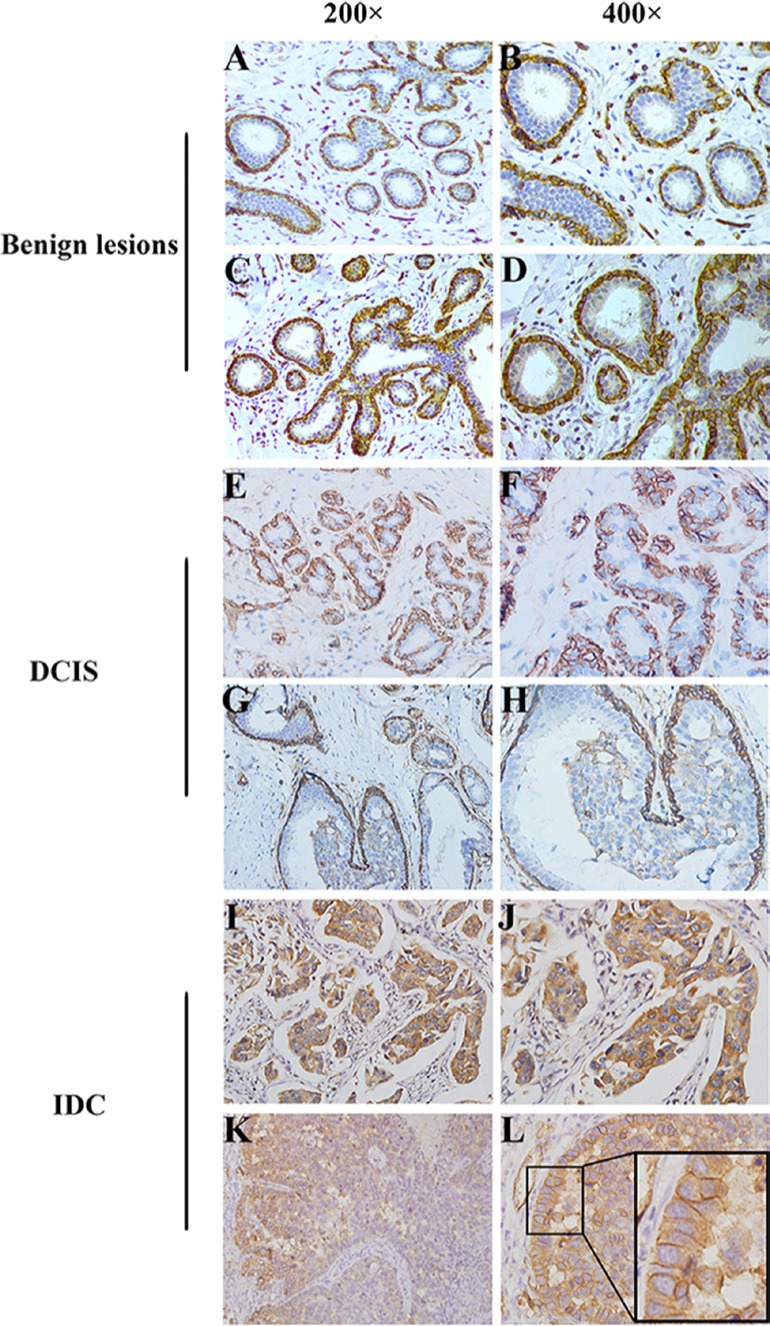
Distinct cellular localization of AQP1 was detected in different breast tissues (**A, B**) There was a strongly positive staining of AQP1 in membrane of myoepithelial cells in breast benign lesions, while no expression of AQP1 was observed in ductal glandular epithelial cells. (**C, D**) There was a strongly positive staining of AQP1 in membrane of myoepithelial cells as well as a weak staining in ductal glandular epithelial cells in breast benign lesions. (**E, F**) There was a strongly positive staining of AQP1 in membrane of myoepithelial cells in DCIS, while no expression of AQP1 was observed in ductal glandular epithelial cells. (**G, H**) There was a strongly positive staining of AQP1 in membrane of myoepithelial cells as well as a weak staining in ductal glandular epithelial cells in DCIS. (**I, J**) High cytoplasmic expression of AQP1 was observed in breast cancer cells in IDC specimens. (**K, L**) Strong membranous expression of AQP1 with an admixture of less intensive cytoplasmic staining for AQP1. The right panel (magnification 400 ×) is the amplification of the left panel (magnification 200 ×).

In the present study, AQP1 expression was evaluated by immunohistochemistry analysis in 341 cases of IDC, 45 cases of DCIS and 33 cases of benign breast lesions. AQP1 was localized dominantly in the membrane of myoepithelial cells of ducts in benign breast lesions and DCIS (Figure 1A–1H). However, a strongly positive staining of AQP1 in the cytoplasm of cancer cells could be observed in IDC (Figure 1I and 1J). We found AQP1 was localized dominantly in the cytoplasm of IDC cells and 77.4% (264/341) cases exhibited merely cytoplasmic expression of AQP1. Very few IDC cases (5.0%, 17/341) showed strong membranous expression of AQP1 with an admixture of less intensive cytoplasmic staining (Figure 1K and 1L) which were ignored in the following studies in order to focus on the role of cytoplasm AQP1 expression in tumor progression.

The intensity of cytoplasmic AQP1 staining was shown in representative images as Supplementary Figure S1. We found the cytoplasmic expression of AQP1 in ductal epithelial cells was gradually up-regulated from benign breast lesions to DCIS (*P =* 0.011), and to IDC (*P =* 0.025). 41.9% (143/341) of IDC, 28.9% (13/45) of DCIS and 15.2% (5/33) of benign breast lesions showed high cytoplasmic expression of AQP1, indicating that cytoplasmic AQP1 probably be involved in breast cancer progression (Table 1).

**Table 1:** Cytoplasmic AQP1 expression in glandular epithelium of different breast tissues

Histological type	Cases	AQP1 score, *n* (%)			χ^2^	*P*
		0	1–2	3–9		
Benign lesions	33	21 (63.6)	7 (21.2)	5 (15.2)	23.657	0.000^c^
DCIS^a^	45	14 (31.1)	18 (40.0)	13 (28.9)		
IDC^b^	341	77 (22.6)	121 (35.5)	143 (41.9)		

a DCIS: ductal carcinoma *in situ*.

b IDC: invasive ductal carcinoma.

c *P* value was calculated by Kruskal-Wallis test.

The sequence of the 3 groups: benign breast lesions < DCIS (Z = −2.528, *P* = 0.011) < IDC (Z = −2.243, *P* = 0.025) (Mann-Whitney *U* test).

### Cytoplasmic expression of AQP1 was positively correlated with advanced pathological features of IDC

**Figure 2:**
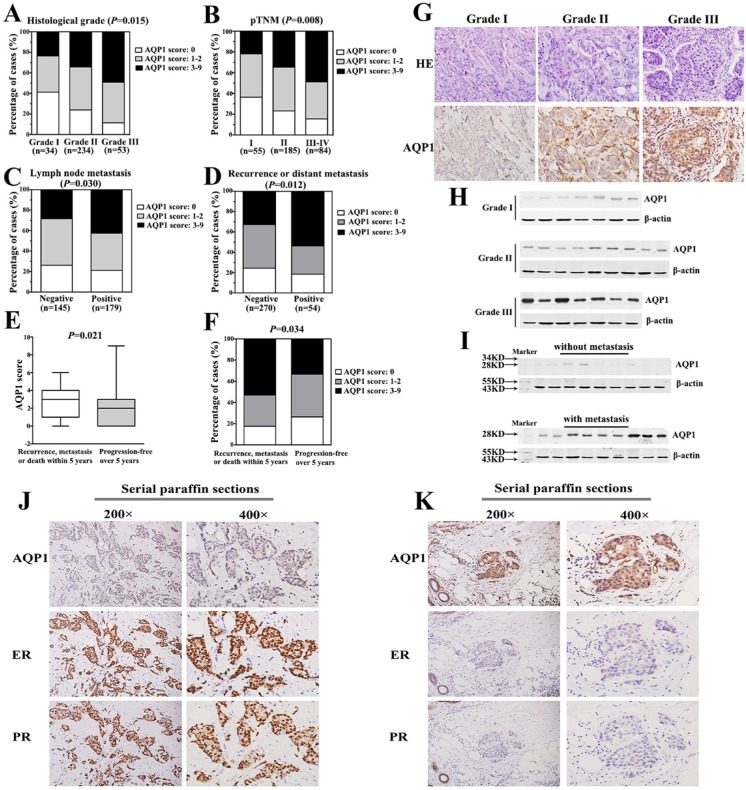
Cytoplasmic expression of AQP1 was positively correlated with breast cancer progression and negatively correlated with ER and PR status (**A**) Cytoplasmic expression of AQP1 increased significantly with increasing histological grade of breast cancer. (**B**) Cytoplasmic expression of AQP1 increased significantly as pTNM stage increased. (**C**) Cytoplasmic expression of AQP1 was higher in patients with lymph node metastasis than that in patients without lymph node metastasis. (**D**) Cytoplasmic expression of AQP1 in patients developing recurrence or distant metastasis was significantly higher than that in patients without recurrence or distant metastasis during follow-up period. (**E**) Cytoplasmic expression of AQP1 in patients who developed metastasis, recurrence or death within 5 years (median score: 3) was higher than that in patients who were disease-free over 5 years (median score: 2) (Mann-Whitney *U* test, *P =* 0.021). (**F**) 52.9% (27/51) of patients who developed metastasis, recurrence or death within 5 years showed high expression of AQP1, while 33.2% (64/193) of patients who were disease-free over 5 years exhibited high expression of AQP1 (χ^2^ test, *P =* 0.034). (**G**) Representative images of AQP1 expression in breast cancer specimens with different histological grades (magnification 200 ×). Upper part: hematoxylin-eosin (H & E) staining; Lower part: immunohistochemical staining. (**H**) Western blot analysis of AQP1 expression in 23 cases of frozen breast tumor specimens (Grade I: 7 cases, Grade II: 9 cases, Grade III: 7 cases). β-actin was used as a loading control. (**I**) Western blot analysis of AQP1 expression in frozen primary tumor tissues. Upper panel: 9 cases without metastasis at the diagnosis time. Lower panel: 9 cases with lymph node metastasis at the diagnosis time. β-actin was used as a loading control. (**J, K**) Cytoplasmic expression of AQP1 was negatively correlated with ER and PR. The expression of AQP1, ER and PR were detected using serial paraffin sections by immunohistochemistry analysis. The right panel (magnification 400 ×) is the amplification of the left panel (magnification 200 ×).

Expression of AQP1 was positively correlated with histological grade, tumor size, pTNM stage, lymph node metastasis and recurrence or distant metastasis (Table 2). Cytoplasmic expression of AQP1 increased with increasing histological grade and pTNM stage of breast cancer (Figure 2A and 2B). Cytoplasmic expression of AQP1 was higher in patients with lymph node metastasis than that in patients without lymph node metastasis (Figure 2C). The similar tendency was observed in patients with recurrence or distant metastasis and patients without recurrence or distant metastasis (Figure 2D). Additionally, we found cytoplasmic expression of AQP1 in patients developing metastasis, recurrence or death within 5 years was higher than that in patients who were disease-free over 5 years (*P =* 0.021, Figure 2E). 52.9% (27/51) of patients who developed metastasis, recurrence or death within 5 years showed high cytoplasmic expression of AQP1, while 33.2% (64/193) of patients in the disease-free over 5 years group exhibited high cytoplasmic expression of AQP1 (Figure 2F). Representative immunohistochemical images of AQP1 expression in Figure 2G showed that there was a dramatic up-regulation of AQP1 expression as histological grade increased. It was also confirmed by Western blot analysis in Figure 2H and Supplementary Figure S2. The detailed information of patients in Figure 2H was shown in Supplementary Table S1. AQP1 expression in patients with lymph node metastasis was higher than that in patients without metastasis (Figure 2I).

**Table 2:** Cytoplasmic AQP1 expression in IDC patients

Pathological features	Cases	AQP1 score, *n* (%)			*r*_s_	*P*
		0	1–2	3–9		
Age, y					0.082	0.139
< 50	146	41 (28.1)	56 (38.3)	49 (33.6)		
≥ 50	178	35 (19.7)	75 (42.1)	68 (38.2)		
Histological grade^a^					0.186	0.001
I	34	14 (41.2)	12 (35.3)	8 (23.5)		
II	234	56 (23.9)	98 (41.9)	80 (34.2)		
III	53	6 (11.3)	21 (39.6)	26 (49.1)		
Tumor size, cm					0.130	0.020
≤ 2	90	30 (33.3)	37 (41.1)	23 (25.6)		
2–5	214	40 (18.7)	86 (40.2)	88 (41.1)		
> 5	20	6 (30.0)	8 (40.0)	6 (30.0)		
Lymph node metastasis					0.129	0.020
Negative	145	38 (26.2)	66 (45.5)	41 (28.3)		
Positive	179	38 (21.2)	65 (36.3)	76 (42.5)		
pTNM					0.202	0.000
I	55	20 (36.4)	23 (41.8)	12 (21.8)		
II	185	43 (23.2)	78 (42.2)	64 (34.6)		
III–IV	84	13 (15.5)	30 (35.7)	41 (48.8)		
Recurrence or distant metastasis					0.138	0.013
NO	270	66 (24.4)	116 (43.0)	88 (32.6)		
Yes	54	10 (18.5)	15 (27.8)	29 (53.7)		
ER status^a^					−0.120	0.031
Negative	92	14 (15.2)	39 (42.4)	39 (42.4)		
Positive	231	62 (26.8)	92 (39.8)	77 (33.3)		
PR status^a^					−0.159	0.004
Negative	68	11 (16.2)	22 (32.3)	35 (51.5)		
Positive	255	65 (25.5)	109 (42.7)	81 (31.8)		
Her2 status^a^					0.075	0.178
– ∼ +	252	60 (23.8)	108 (42.9)	84 (33.3)		
++ ∼ +++	71	16 (22.5)	23 (32.4)	32 (45.1)		

a Some missing data

It was worth noting that cytoplasmic expression of AQP1 was negatively correlated with the expression of ER (*r*_s_ = −0.120, *P =* 0.031) and PR (*r*_s_ = −0.159, *P =* 0.004) (Table 2). It was further validated by immunohistochemistry analysis using serial pathological sections and the representative images of AQP1, ER and PR expression in the same visual field of corresponding parts were shown in Figure 2J and 2K.

### Cytoplasmic expression of AQP1 in lymph node metastases was higher than their paired primary tumors

**Figure 3:**
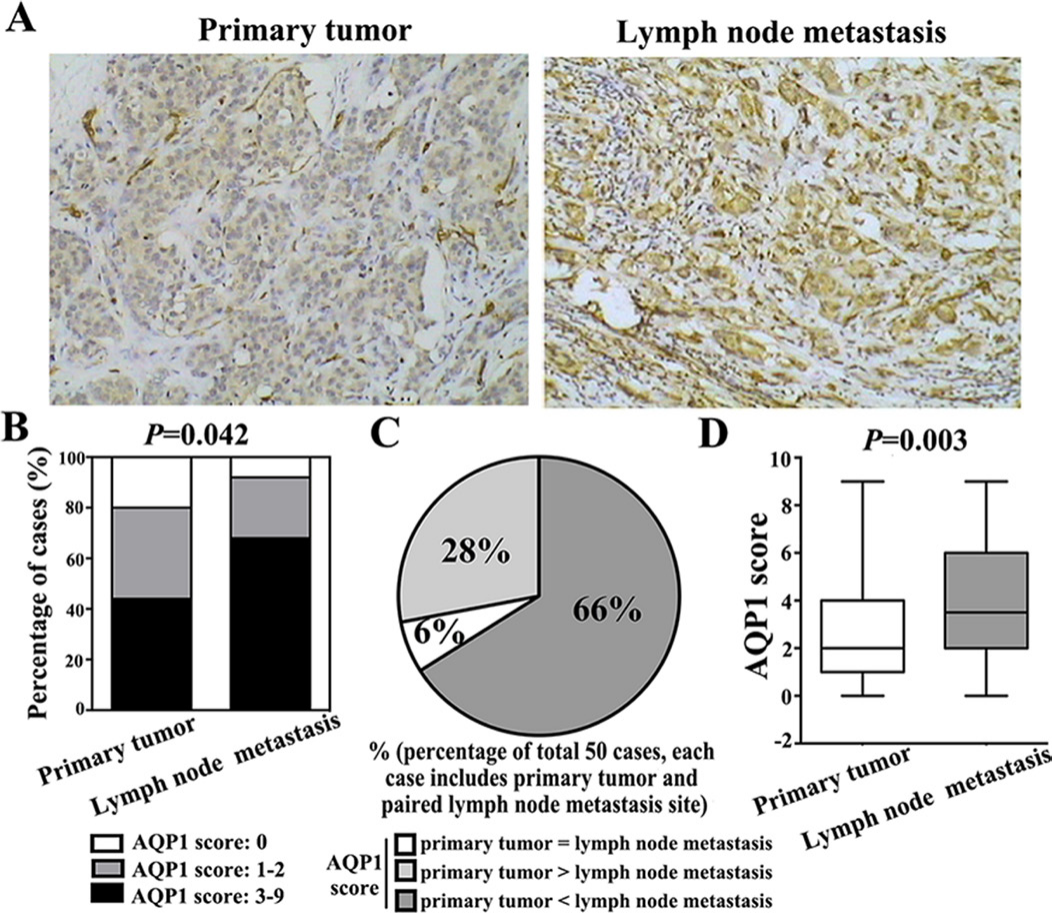
Cytoplasmic expression of AQP1 in lymph node metastases was higher than their paired primary tumors in total 50 paired cases (**A**) Representative immunohistochemical images of AQP1 expression in primary breast cancer and paired lymph node metastasis (magnification 200 ×). (**B**) 68% (34/50) lymph node metastasis specimens exhibited high AQP1 cytoplasmic expression, while 44% (22/50) primary breast cancer specimens showed high AQP1 cytoplasmic expression (χ^2^ = 6.343, *P =* 0.042). (**C**) Among total 50 paired cases (each case including primary tumor and paired lymph node metastasis specimens), 66% (33/50) cases showed that AQP1 expression in lymph node metastases was higher than paired primary tumors, 6% (3/50) cases showed that AQP1 expression in lymph node metastases was similar to their paired primary tumors and 28% (14/50) cases showed that AQP1 expression in lymph node metastasis was lower than their paired primary tumor. (**D**) Cytoplasmic AQP1 expression in primary breast cancer specimens (median score: 2.0) was lower than that in their paired lymph node metastases (median score: 3.5) (Mann-Whitney *U* test, *P =* 0.003).

Based on above results, we analyzed AQP1 cytoplasmic expression in 50 primary breast cancer tissues and their paired lymph node metastases. Expression of AQP1 was higher in lymph node metastases than their paired primary sites (Figure 3A). 44% (22/50) primary breast cancer tissues showed high cytoplasmic AQP1 expression, while 68% (34/50) lymph node metastasis specimens exhibited high AQP1 cytoplasmic expression (*P =* 0.042, Figure 3B). Notably, 66% (33/50) of the total 50 paired cases showed that AQP1 expression in lymph node metastases was higher than paired primary tumors (Figure 3C). Moreover, as shown in Figure 3D, the median score of AQP1 cytoplasmic expression in lymph node metastases was higher than that in primary sites (*P =* 0.003, Figure 3D).

### High cytoplasmic expression of AQP1 indicated worse prognosis of breast cancer patients

**Figure 4:**
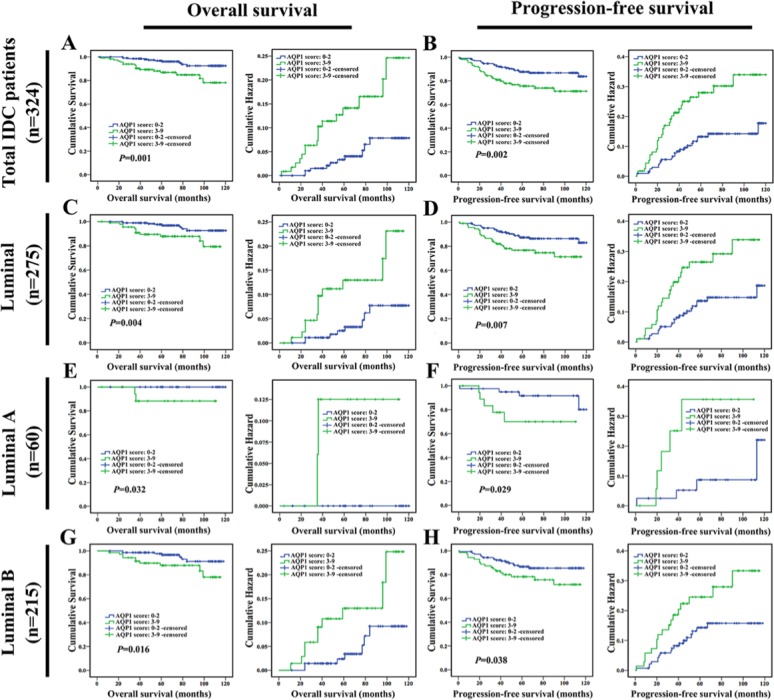
High cytoplasmic expression of AQP1 indicated poor prognosis in breast cancer patients Overall survival (OS) and progression-free survival (PFS) curves of total 324 IDC patients were shown in (**A** and **B**) respectively. OS and PFS of 275 luminal cases were shown in (**C** and **D**) respectively. OS and PFS of 60 luminal A cases were shown in (**E** and **F**) respectively. OS and PFS of 215 luminal B cases were shown in (**G** and **H**) respectively.

In order to explore the role of AQP1 in breast cancer prognosis, we analyzed 324 IDC patients with complete clinical follow-up. 4.8% (10/207) patients in the low AQP1 expression group died of tumor; while 14.5% (17/117) patients in the high expression group suffered tumor-related death. Moreover, patients with high level of AQP1 showed shorter overall survival (OS) (*P =* 0.001, Figure 4A) and progression-free survival (PFS) (*P* = 0.002, Figure 4B). The corresponding hazard curves were depicted in Figure 4A and 4B.

To further assess the independent prognostic value, the possible impact of patients, tumor variables were investigated by univariate analysis with respect to OS and PFS (Table 3). High cytoplasmic expression of AQP1 indicated shorter cancer-specific OS and PFS. In multivariate Cox regression analysis, high cytoplasmic expression of AQP1 was proved to be an independent prognostic factor (Table 3).

**Table 3:** Univariate and multivariate analysis for overall survival (OS) and progression-free survival (PFS)

Variables	OS (univariate)		OS (multivariate)		PFS (univariate)		PFS (multivariate)	
	HR (95% CI)	*P*	HR (95% CI)	*P*	HR (95% CI)	*P*	HR (95% CI)	*P*
Age	1.087 (0.508–2.323)	0.830	1.736 (0.581–5.189)	0.323	0.923 (0.541–1.574)	0.768	0.819 (0.356–1.887)	0.639
Menopausal status	0.866 (0.405–1.852)	0.711	0.574 (0.191–1.728)	0.324	1.022 (0.599–1.744)	0.935	1.230 (0.534–2.832)	0.627
Family history	1.460 (0.345–6.175)	0.607	2.087 (0.466–9.352)	0.336	1.639 (0.591–4.543)	0.342	2.121 (0.752–5.978)	0.155
Histological grade	3.331 (1.637–6.780)	0.001	2.961 (1.421–6.169)	0.004	2.146 (1.289–3.573)	0.003	1.868 (1.097–3.180)	0.021
Lymph node status	3.924 (1.486–10.366)	0.006	3.483 (1.264–9.600)	0.016	4.016 (2.021–7.983)	0.000	3.734 (1.851–7.531)	0.000
AQP1 score (0–2 vs. 3–9)	2.968 (1.358–6.486)	0.006	2.439 (1.109–5.366)	0.027	2.090 (1.224–3.569)	0.007	1.740 (1.013–2.989)	0.045

Next, survival analysis was performed in patients with detailed classification according to molecular subtypes of breast cancer. The results indicated that high cytoplasmic expression of AQP1 led to a worse prognosis in 275 cases of luminal breast cancer (including 60 luminal A cases and 215 luminal B cases) (Figure 4C–4H). However, in non-luminal breast cancer including triple-negative subtype, there was no difference between patients with low or high expression of AQP1 (Supplementary Figure S3).

### Overexpression of AQP1 promoted proliferation and invasion of breast cancer cells

**Figure 5:**
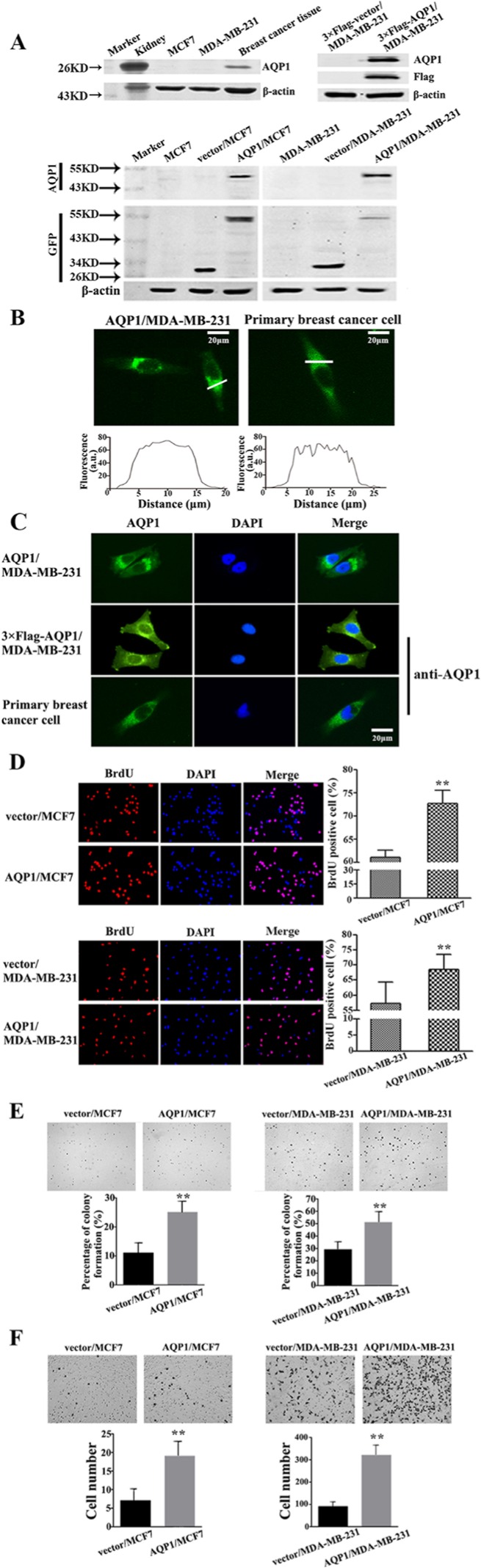
Overexpression of AQP1 promoted proliferation and invasion of breast cancer cells (**A**) Western blot results of exogenous AQP1 expression in MCF7 and MDA-MB-231 cells. AQP1 expression was detected by primary GFP and AQP1 antibodies in AQP1/MCF7 and AQP1/MDA-MB-231 cells, respectively. AQP1 expression was detected by primary Flag and AQP1 antibodies in 3 × Flag-AQP1/MDA-MB-231 cells. β-actin was used as loading controls. Kidney tissue from mouse was used as a positive control. (**B**) Cytoplasmic localization of AQP1 was detected in AQP1-overexpressing MDA-MB-231 cells (left panel, AQP1-GFP fusion protein) and primary breast cancer cells (right panel, anti-AQP1 antibody). Fluorescence amplitudes (a.u., arbitrary units) along the line scans (in white on the image) were displayed graphically below each image. (**C**) Immunofluorescence localization analysis of AQP1 in AQP1-overexpressing MDA-MB-231 cells (upper: GFP labeled AQP1-overexpressing cells; middle: 3 × Flag labeled AQP1-overexpressing cells) and primary breast cancer cells (lower). DAPI was used to stain the nuclei. AQP1 expression was analyzed by GFP fluorescence (upper) and AQP1 antibody (middle and lower). (**D**) Proliferation ability was detected by BrdU incorporation analysis in AQP1-overexpressing MCF7 and MDA-MB-231 cells (magnification 200 ×). (**E**) Colony formation assays were performed using AQP1-overexpressing MCF7 and MDA-MB-231 cells (magnification 200 ×). (**F**) Invasion ability was detected using AQP1-overexpressing MCF7 and MDA-MB-231 cells. Bars are mean ± SD. All experiments were performed 3 times independently. (**P < 0.01)

In the following studies, the role of AQP1 in breast cancer development was validated by *in vitro* experiments. Since endogenous AQP1 was undetectable in parental MCF7 and MDA-MB-231 cell lines, we overexpressed AQP1 in them and detected its exogenous expression by Western blot (Figure 5A). In addition, both endogenous expression of AQP1 in primary breast cancer cells and exogenous overexpression of AQP1 in MDA-MB-231 cells were examined by immunofluorescence analyses. Cytoplasmic staining of AQP1 was confirmed in above both types of cells, which was consistent with AQP1 localization in clinical analysis (Figure 5B and 5C). It was reported that expression of AQP1 altered responding to a series of stimuli [23]. We found that EGF (epidermal growth factor) stimulation could induce re-distribution of AQP1 from cytoplasm to cell membrane (Supplementary Figure S4).

Furthermore, the abilities of both proliferation and colony formation increased in both AQP1/MDA-MB-231 and AQP1/MCF7 cells compared with control cells (Figure 5D and 5E). We also examined the invasive ability by Matrigel Boyden chamber assays, in which increased invasion was observed in AQP1-overexpressing cells (Figure 5F).

## DISCUSSION

In our study, no endogenous AQP1 expression was detected in MDA-MB-231 and MCF7 cell lines by Western blot. This loss of AQP1 in long-term tumor cell cultures and cell lines is likely a result of culture condition. In further support of this speculation, reports have shown that AQP1 was regulated via osmotic response elements and hypertonicity. Cells which were not stimulated by constant changes in osmolarity might selectively downregulate AQP1, while AQP1 was upregulated by hypertonic challenge in cells lacking endogenous expression [24–27].

Migration and invasion of tumor cells are crucial steps in tumor progression [28–30]. Verkman's group initially reported the involvement of AQP1 in cell migration [31]. Subsequent studies by Monzani et al. demonstrated that AQP1 was not only a water channel but also a critical scaffold for plasma-membrane associated multiprotein-complex important for cytoskeleton build-up, adhesion and motility [32]. According to the model they proposed, AQP1 bound with Lin7 (a plasma membrane-associated cytoplasmic protein required for the organization of cytoskeleton), affected the organization of the cytoskeleton and contributed to cell migration through Lin7/β-catenin interaction. β-catenin in nuclei could act as a transcriptional coactivator binding with the members of the T cell factor/lymphoid enhancer factor (TCF/LEF) transcription factor family whose target genes include matrix metalloproteinases, chemokines or cytoskeletal proteins, which regulate cell migration and cancer invasion [32]. In addition, a recent study indicated that AQP1 enhanced migration of bone marrow mesenchymal stem cells (MSC) through modulating the expression of β-catenin and FAK, both of which were co-immunoprecipitated with AQP1. FAK is crucial for migrating and the depletion of AQP1 led to the degradation of FAK which abolished the promotion effects of AQP1 on migration [33]. Moreover, AQP1 also co-localized with ezrin (a cytoskeletal protein that crosslink the actin cytoskeleton and plasma membrane) and knockdown of AQP1 could significantly inhibit cell migration and invasion [34]. In line with previous studies, our studies demonstrated that overexpression of AQP1 in breast cancer cells induced an increased migration and invasion, which were also consistent with our present clinical findings.

Previous reports have demonstrated that AQP1 also acts as an important player in cell proliferation [35–37]. Both results of BrdU and soft agar assays in our present study showed that overexpression of AQP1 promoted the proliferation of breast cancer cells, which were consistent with previous reports and our present clinical findings.

In conclusion, our study provided the first evidence that cytoplasmic expression of AQP1 promoted breast cancer progression and it could be a potential prognostic biomarker for breast cancer.

## MATERIALS AND METHODS

### Ethical statement

This study was reviewed and approved by the Ethic Committee of Tianjin Medical University Cancer Institute & Hospital. All experiments were performed in accordance with relevant guidelines and regulations of Ethic Committee of Tianjin Medical University Cancer Institute & Hospital. All the patients signed an informed consent for participation of the study and the use of their biological tissues prior to surgery.

### Patient selection and clinical information

Paraffin-embedded specimens from 341 breast cancer patients with IDC, diagnosed between 2004 and 2007, together with 45 cases of DCIS and 33 cases of benign breast lesions were reviewed and selected from the archives of the Department of Breast Cancer Pathology and Research Laboratory, Tianjin Medical University Cancer Institute & Hospital (Tianjin, China). The histopathology was reviewed and the diagnosis of each case was confirmed independently by two pathologists according to World Health Organization (WHO) criteria. None of them had received neo-adjuvant chemotherapy or preoperative radiation therapy.

In this study, we found that AQP1 was localized dominantly in the cytoplasm of cancer cells in total 341 IDC specimens. Only 5% cases (17/341) exhibited a strong membranous expression of AQP1, together with an admixture of less intensive cytoplasmic staining. Therefore, the rest 324 patients were regarded as our research targets in our study. The mean age of the 324 IDC patients was 51.51 years old (range, 27–89). The patients were followed up for 2–120 months during which 14 (4.3%) patients suffered local or regional tumor recurrence, 40 (12.3%) patients developed distant metastasis, and 27 (8.3%) patients died of tumors. In addition, 244 patients were followed up more than 60 months and 51 patients suffered disease progression (recurrence, metastasis or death) within 5 years.

### Immunohistochemistry and scoring

IHC for AQP1 was performed using standard techniques by S-P method. Antigen retrieval was performed at 121°C for 2 minutes 15 seconds by citrate buffer. After serial blocking with hydrogen peroxide and normal goat serum, the sections were incubated with primary antibody against AQP1 (1:100, SC-20810, Santa Cruz, CA, USA) overnight at 4°C. Sections were incubated sequentially with biotinylated goat anti-rabbit immunoglobulin and peroxidase-conjugated streptavidin. The enzyme substrate was 3, 3′-diaminobenzidine tetra-hydrochloride (DAB).

### Evaluation of staining

Evaluation of immunostaining by IHC score was based on a double scoring system (staining intensity multiplied by staining area), producing a total range of 0 to 9. Staining intensity was scored as follows: 0 (−) no staining, 1 (+) definite but weak staining, 2 (++) moderate staining and 3 (+++) intense staining. The staining area was scored as follows: 0 (no staining of cells in any microscopic field), 1 (1–49% of cells stained positive), 2 (50–75% of cells stained positive) and 3 (76–100% of cells stained positive). Patients were categorized into 3 groups according to IHC score of AQP1: AQP1 score (0), AQP1 score (1–2) and AQP1 score (3–9). Additionally, AQP1 score (0–2) was defined as low expression and AQP1 score (3–9) was defined as high expression.

### Cell culture and reagents

A detailed experimental procedure for primary breast cancer cells was described in the study of Kobayashi et al. [38]. In brief, cancer tissues from patients diagnosed with invasive ductal breast carcinoma, not-otherwise type (IDC-NOS), were harvested. Each fresh breast tumor specimen was digested in dispersion collagenase enzyme and the dispersed cancer cells were incubated in a collagen-coated flask. The viable cells alone adhering to the collagen gel layer were then collected and added to reconstructed Type I collagen solution (Cellmatrix type CD^™^; Niita Gelatin Inc., Yao, Japan).

MDA-MB-231 and MCF7 cell lines were obtained from the American Type Culture Collection (Manassas, VA, USA). They were cultured in DMEM medium supplemented with 10% fetal bovine serum (FBS) in a 5% CO_2_ incubator at 37°C. Cells had been tested and authenticated by DNA (STR) profiling, work performed by Beijing Microread Genetics Co., Ltd. (Beijing, China).

### Construction of lentiviral vector expressed with full length of AQP1

Full length of AQP1 was amplified by PCR using primers for human AQP1 (GenBank accession No. NM_198098.2, Forward: 5′-AATTGAATTCGCCAC CATGGCCAGCGAGTTCAAG-3′ and Reverse: 5′-CGGGATCCCTATTTGGGCTTCATCTC-3′). AQP1 with GFP label and AQP1 with 3 × Flag tag were cloned into pCDH-CMV-MCS-EF1-Puro lentiviral vector (http://www.addgene.org/) respectively. The sequences of the inserts were 100% correct.

### Lentivirus production and infection

Lentiviruses were produced by co-transfection of lentiviral plasmid, packing plasmids ΔR and pVSVg into HEK-293T cells. After transfection, supernatant was collected and the virus was used to infect cells. Lentivirus-infected cells were screened by 2 μg/ml puromycin for 2 weeks to establish stably expressing cells and verified by Western blot analysis.

### Western blot

Tissues or cells were lysed with SDS lysis buffer on ice directly. Equal amounts of proteins were separated by SDS-PAGE and electrotransferred onto nitrocellulose membranes. The blots were incubated by a primary antibody: AQP1, GFP (KM8009, SanJian, China), Flag (AF519–1, Beyotime, China) and β-actin (SC-47778, Santa Cruz, USA). The membrane was then treated with secondary antibodies.

### Immunofluorescence analyses and determination of subcellular localization

Cells (1 × 10^5^ cells/well) were seeded in 35-mm dishes. After 24 h, they were fixed, permeabilized, blocked with 3% BSA and incubated with primary AQP1 antibody (1:100) at 4°C overnight. Then the cells were incubated with Alexa fluor 488-conjugated goat anti-rabbit IgG for 2 hours in dark. DAPI (4′,6-diamidino-2-phenylindole) was used to stain nuclei. Images were acquired using a fluorescence microscope (magnification 200 ×). The fluorescence intensity over the distance (covering cell membrane and cytoplasm but avoiding nucleus) was measured according to the previous report [21].

### Proliferation assay

Cells (1 × 10^5^ cells/well) were cultured in 35-mm dishes at 37°C. 1 mg/ml BrdU (5-bromo-2′-deoxyuridine) was added into each dish. After 48 h, cells were fixed and incorporated BrdU was detected and quantified. Cells were also stained with DAPI. Both BrdU and DAPI positive cells in five random fields were counted.

### Colony formation assay

1 × 10^4^ cells were mixed with DMEM supplemented with 10% FBS and 0.35% agarose and plated on top of a solidified layer of agarose. After 3 weeks of incubation, colonies were stained with 0.005% crystal violet. Colonies larger than 50 μm were scored and photographed using an Olympus microscope (Olympus, Japan).

### Matrigel invasion assay

Boyden chamber invasion assays were performed to measure cell invasion *in vitro*. Briefly, cells were added in upper wells and binding medium with 10 ng/ml of EGF was added to the lower wells. After 24 h of incubation, the invading cells were fixed, stained, counted and photographed under a microscope in five pre-determined fields at 200 × magnifications.

### Statistical analyses

The SPSS 17.0 software package was used for statistical analysis. Mann-Whitney *U* test, Kruskal-Wallis test, ANOVA test, and χ^2^ test were performed for group comparisons and correlations between two variables were evaluated by Spearman's Rank-Correlation test. Overall survival (OS) and progression-free survival (PFS) rates were estimated using the Kaplan-Meier method, and the log-rank test was applied to compute *P* values. The Cox proportional hazards regression model was performed toward the identification of relevant prognostic factors. For *in vitro* work, statistical significance for comparisons between groups was determined using a two-tailed Student's *t*-test. All data was presented as mean ± standard deviation. A two-sided *P* < 0.05 was considered statistically significant in all analyses.

## SUPPLEMENTARY MATERIALS TABLE AND FIGURES


