# Supplementary material for: Expression of aquaporin1, a water channel protein, in cytoplasm is negatively correlated with prognosis of breast cancer patients
Source: Oncotarget. 2016 Jan 23;7(7):8143–54. doi: 10.18632/oncotarget.6994 (PMC4884982; doi:10.18632/oncotarget.6994)
Supplement: Supplementary file 1 [file oncotarget-07-8143-s001.pdf]

Expression of aquaporin1, a water channel protein, in cytoplasm is negatively correlated with prognosis of breast cancer patients

Supplementary Materials

Supplementary Table S1: Information of patients in figure 2H

Number*	Age (years)	Histological grade	ER (%)	PR (%)	Her2
1	73	I	90	40	+
2	43	I	75	80	+
3	44	I	90	90	–
4	55	I	90	70	+
5	50	I	90	90	–
6	63	I	85	8	+
7	67	I	90	65	+
8	71	II	90	70	+
9	32	II	90	80	++
10	51	II	< 1	< 1	–
11	64	II	90	90	++
12	52	II	80	50	+
13	61	II	90	< 1	++
14	59	II	90	85	++
15	47	II	50	60	++
16	64	II	< 1	< 1	++
17	63	III	< 1	< 1	++
18	50	III	< 1	< 1	++
19	46	III	< 1	< 1	+++
20	58	III	< 1	< 1	–
21	75	III	90	20	++
22	47	III	60	60	+
23	39	III	< 1	< 1	+++

*The order of specimens was same as Western blot analysis of Figure 2H.

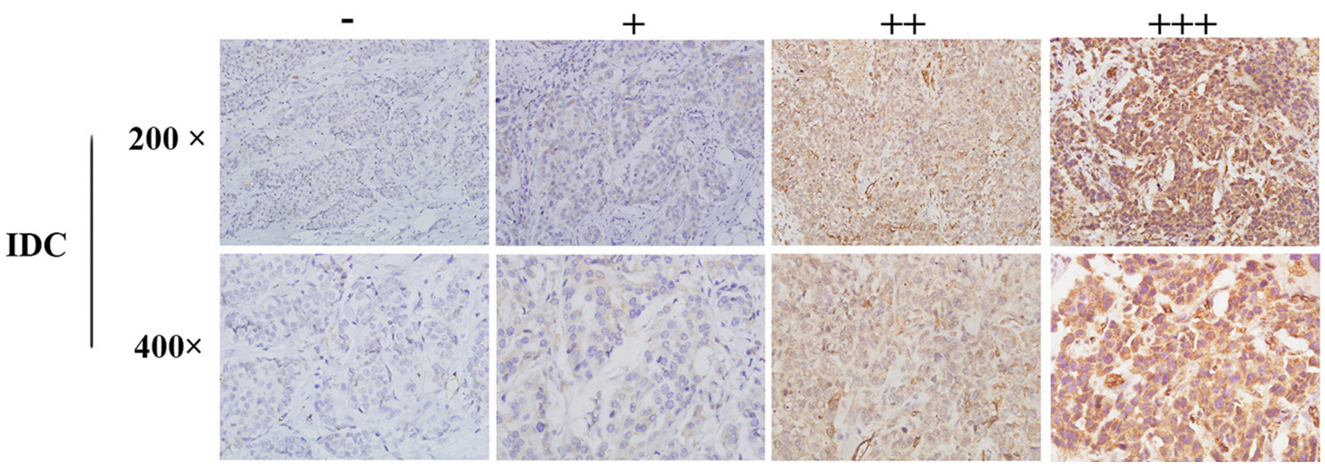

Supplementary Figure S1: Varying staining intensity of AQP1 protein in IDC breast tissues: (–) no staining; (+) definite but weak staining; (++) moderate staining and (+++) strong staining. The lower part (magnification 400 ×) is the amplification of the upper part (magnification 200 ×).

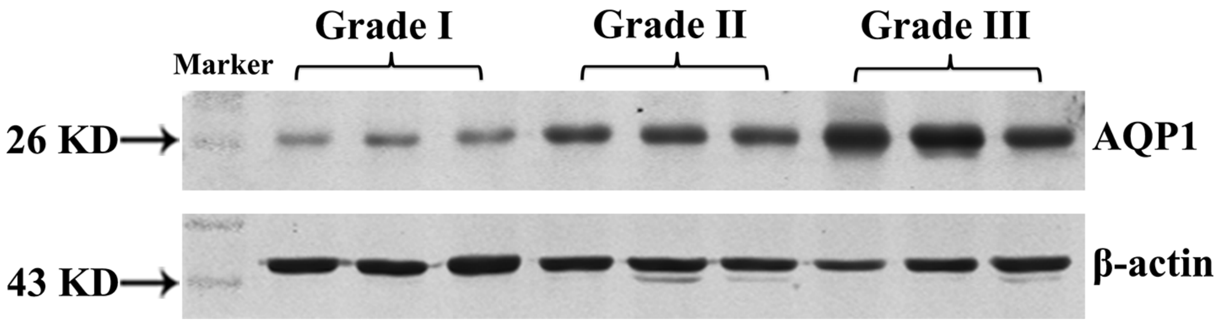

Supplementary Figure S2: Western blot result of AQP1 protein expression in IDC specimens with different pathological grades in one gel. All the patients were female (Grade I: 3 cases; Grade II: 3 cases; Grade III: 3 cases). β-actin was used as a loading control.

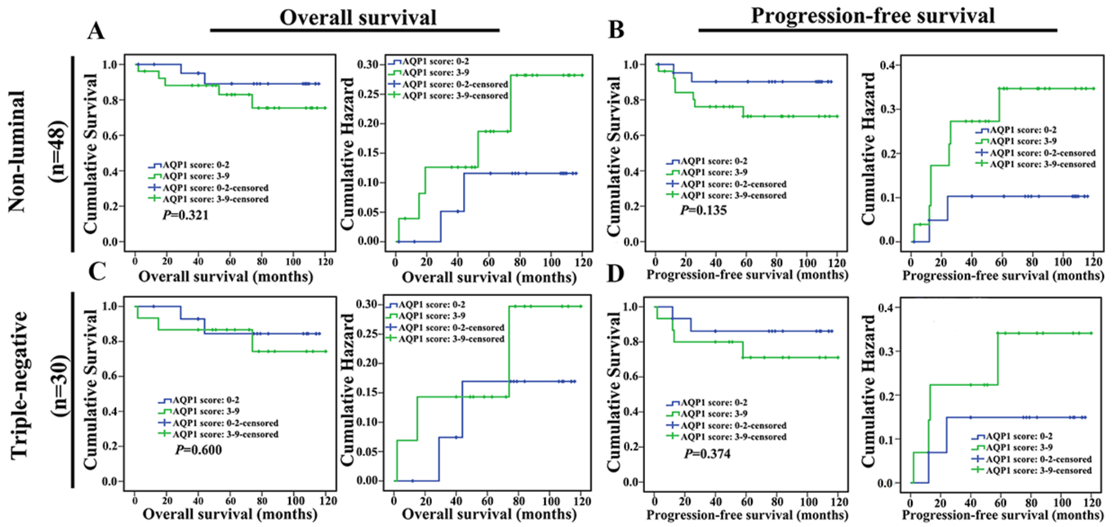

Supplementary Figure S3: Cytoplasmic expression of AQP1 was not associated with prognosis of non-luminal patients or triple-negative patients. (**A**) There was no difference between OS of non-luminal patients with high AQP1 expression and that of patients with low AQP1 expression (P = 0.321). (**B**) There was no difference between PFS of non-luminal patients with high AQP1 expression and that of patients with low AQP1 expression (P = 0.135). (**C**) There was no difference between OS of triple-negative patients with high AQP1 expression and that of patients with low AQP1 expression (P = 0.600). (**D**) There was no difference between PFS of triple-negative patients with high AQP1 expression and that of patients with low AQP1 expression (P = 0.374).

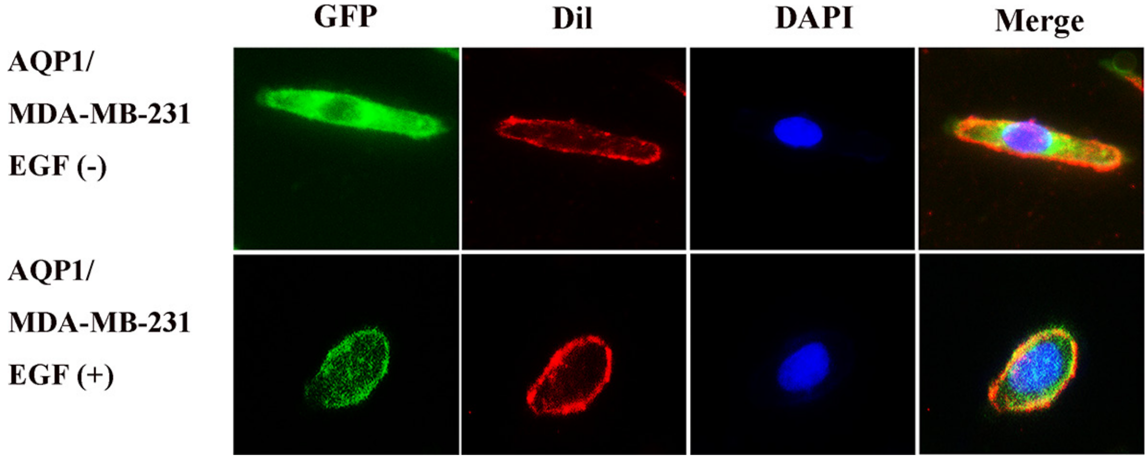

Supplementary Figure S4: EGF (epidermal growth factor) stimulation-induced AQP1 subcellular translocation in GFP labeled fusion protein AQP1-overexpressing MDA-MB-231 cells. AQP1 translocation from cytoplasm to membrane was triggered by EGF treatment (10 ng/ml, 20 min). Nuclei were stained with DAPI (blue color). Cell membrane was stained with Dil (red color) (1, 1-dioctadecyl-3, 3,3, 3-tetramethylindocarbocyanine perchlorate). (Magnification 200 ×).
